# Methyl *N*-(4-chloro­phen­yl)carbamate

**DOI:** 10.1107/S1600536811037123

**Published:** 2011-09-30

**Authors:** Yu-Feng Li

**Affiliations:** a Microscale Science Institute, Department of Chemistry and Chemical Engineering, Weifang University, Weifang 261061, People’s Republic of China

## Abstract

In the title compound, C_8_H_8_ClNO_2_, the dihedral angle between the chloro­benzene ring and the side chain is 8.79 (11)°. In the crystal, mol­ecules are linked by N—H⋯O hydrogen bonds into a *C*(4) chain propagating in the *b*-axis direction.

## Related literature

For related structures, see: Li (2011*a* ▶,*b* ▶).
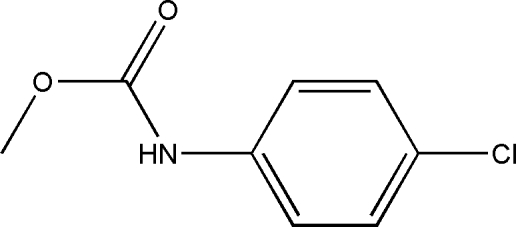


## Experimental

### 

#### Crystal data

C_8_H_8_ClNO_2_
*M* *_r_* = 185.60
Monoclinic, 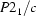

*a* = 11.126 (2) Å
*b* = 9.833 (2) Å
*c* = 8.0076 (16) Å
β = 99.34 (3)°
*V* = 864.5 (3) Å^3^
*Z* = 4
Mo *K*α radiation
μ = 0.40 mm^−1^
*T* = 293 K
0.23 × 0.20 × 0.18 mm

#### Data collection

Bruker SMART CCD diffractometer
8281 measured reflections
1987 independent reflections
1011 reflections with *I* > 2σ(*I*)
*R*_int_ = 0.036

#### Refinement

*R*[*F* ^2^ > 2σ(*F* ^2^)] = 0.041
*wR*(*F* ^2^) = 0.159
*S* = 1.06
1987 reflections
109 parameters
H-atom parameters constrained
Δρ_max_ = 0.18 e Å^−3^
Δρ_min_ = −0.26 e Å^−3^

### 

Data collection: *SMART* (Bruker, 1997 ▶); cell refinement: *SAINT* (Bruker, 1997 ▶); data reduction: *SAINT*; program(s) used to solve structure: *SHELXS97* (Sheldrick, 2008 ▶); program(s) used to refine structure: *SHELXL97* (Sheldrick, 2008 ▶); molecular graphics: *SHELXTL* (Sheldrick, 2008 ▶); software used to prepare material for publication: *SHELXTL*.

## Supplementary Material

Crystal structure: contains datablock(s) global, I. DOI: 10.1107/S1600536811037123/hb6394sup1.cif

Structure factors: contains datablock(s) I. DOI: 10.1107/S1600536811037123/hb6394Isup2.hkl

Supplementary material file. DOI: 10.1107/S1600536811037123/hb6394Isup3.cml

Additional supplementary materials:  crystallographic information; 3D view; checkCIF report

## Figures and Tables

**Table 1:** Hydrogen-bond geometry (Å, °)

*D*—H⋯*A*	*D*—H	H⋯*A*	*D*⋯*A*	*D*—H⋯*A*
N1—H1*A*⋯O2^i^	0.86	2.22	3.069 (2)	168

Symmetry code: (i) 
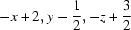
.

**Fig. 1.**
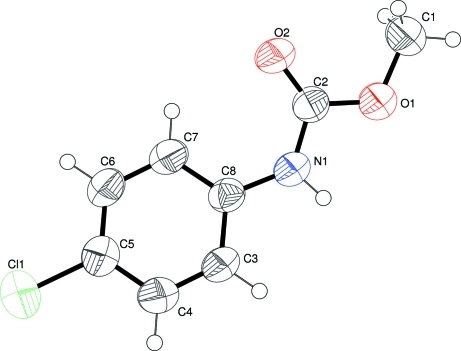
The molecular structure of the title compound showing 50% probability displacement ellipsoids.

## supplementary crystallographic information

### Experimental

A mixture of methanol (0.06 mol), and (4-chlorophenyl)carbamic chloride (0.06 mol) was stirred in refluxing ethanol (15 ml) for 4 h to afford the title compound (0.05 mol, yield 83%). Colourless blocks of the title compound were obtained by recrystallization from ethanol at room temperature.

### Refinement

H atoms were fixed geometrically and allowed to ride on their attached atoms, with C—H distances = 0.93–0.97 Å; N—H = 0.86Å and with *U*_iso_(H) = 1.2*U*_eq_(C,*N*) or 1.5*U*_eq_(C_methyl_).

### Crystal data

**Table:**

C_8_H_8_ClNO_2_	*F*(000) = 384
*M**_r_* = 185.60	*D*_x_ = 1.426 Mg m^−^^3^
Monoclinic, *P*2_1_/*c*	Mo *K*α radiation, λ = 0.71073 Å
Hall symbol: -P 2ybc	Cell parameters from 1987 reflections
*a* = 11.126 (2) Å	θ = 3.0–27.2°
*b* = 9.833 (2) Å	µ = 0.40 mm^−^^1^
*c* = 8.0076 (16) Å	*T* = 293 K
β = 99.34 (3)°	Block, colorless
*V* = 864.5 (3) Å^3^	0.23 × 0.20 × 0.18 mm
*Z* = 4	

### Data collection

**Table:**

Bruker SMART CCD diffractometer	1011 reflections with *I* > 2σ(*I*)
Radiation source: fine-focus sealed tube	*R*_int_ = 0.036
graphite	θ_max_ = 27.5°, θ_min_ = 3.3°
φ and ω scans	*h* = −14→13
8281 measured reflections	*k* = −12→12
1987 independent reflections	*l* = −10→10

### Refinement

**Table:**

Refinement on *F*^2^	Primary atom site location: structure-invariant direct methods
Least-squares matrix: full	Secondary atom site location: difference Fourier map
*R*[*F*^2^ > 2σ(*F*^2^)] = 0.041	Hydrogen site location: inferred from neighbouring sites
*wR*(*F*^2^) = 0.159	H-atom parameters constrained
*S* = 1.06	*w* = 1/[σ^2^(*F*_o_^2^) + (0.081*P*)^2^ + 0.0499*P*] where *P* = (*F*_o_^2^ + 2*F*_c_^2^)/3
1987 reflections	(Δ/σ)_max_ < 0.001
109 parameters	Δρ_max_ = 0.18 e Å^−^^3^
0 restraints	Δρ_min_ = −0.26 e Å^−^^3^

### Special details

**Table:**

Geometry. All e.s.d.'s (except the e.s.d. in the dihedral angle between two l.s. planes) are estimated using the full covariance matrix. The cell e.s.d.'s are taken into account individually in the estimation of e.s.d.'s in distances, angles and torsion angles; correlations between e.s.d.'s in cell parameters are only used when they are defined by crystal symmetry. An approximate (isotropic) treatment of cell e.s.d.'s is used for estimating e.s.d.'s involving l.s. planes.
Refinement. Refinement of *F*^2^ against ALL reflections. The weighted *R*-factor *wR* and goodness of fit *S* are based on *F*^2^, conventional *R*-factors *R* are based on *F*, with *F* set to zero for negative *F*^2^. The threshold expression of *F*^2^ > σ(*F*^2^) is used only for calculating *R*-factors(gt) *etc*. and is not relevant to the choice of reflections for refinement. *R*-factors based on *F*^2^ are statistically about twice as large as those based on *F*, and *R*- factors based on ALL data will be even larger.

### Fractional atomic coordinates and isotropic or equivalent isotropic displacement parameters (Å^2^)

**Table:**

	*x*	*y*	*z*	*U*_iso_*/*U*_eq_	
Cl1	0.51185 (8)	0.66723 (9)	0.21431 (13)	0.1092 (4)	
N1	0.95328 (17)	0.52547 (19)	0.6982 (3)	0.0611 (5)	
H1A	0.9543	0.4420	0.7304	0.073*	
O2	1.06775 (16)	0.71949 (15)	0.7364 (2)	0.0699 (5)	
C8	0.8511 (2)	0.56561 (19)	0.5821 (3)	0.0527 (6)	
O1	1.12660 (16)	0.52441 (16)	0.8735 (2)	0.0749 (5)	
C2	1.0498 (2)	0.6012 (2)	0.7656 (3)	0.0570 (6)	
C7	0.8409 (2)	0.6894 (2)	0.4978 (3)	0.0628 (6)	
H7A	0.9042	0.7521	0.5166	0.075*	
C6	0.7360 (2)	0.7191 (2)	0.3855 (3)	0.0660 (7)	
H6A	0.7294	0.8021	0.3290	0.079*	
C5	0.6422 (2)	0.6283 (2)	0.3568 (3)	0.0665 (7)	
C4	0.6513 (2)	0.5048 (2)	0.4395 (3)	0.0715 (7)	
H4A	0.5878	0.4423	0.4195	0.086*	
C3	0.7548 (2)	0.4750 (2)	0.5514 (3)	0.0644 (6)	
H3A	0.7604	0.3920	0.6079	0.077*	
C1	1.2359 (3)	0.5899 (3)	0.9546 (4)	0.0819 (8)	
H1B	1.2841	0.5264	1.0282	0.123*	
H1C	1.2151	0.6659	1.0196	0.123*	
H1D	1.2817	0.6212	0.8703	0.123*	

### Atomic displacement parameters (Å^2^)

**Table:**

	*U*^11^	*U*^22^	*U*^33^	*U*^12^	*U*^13^	*U*^23^
Cl1	0.0837 (6)	0.1053 (7)	0.1253 (8)	−0.0005 (4)	−0.0225 (5)	0.0310 (5)
N1	0.0645 (12)	0.0440 (10)	0.0726 (14)	−0.0029 (8)	0.0044 (10)	0.0032 (8)
O2	0.0731 (11)	0.0436 (9)	0.0893 (13)	−0.0026 (7)	0.0018 (9)	−0.0033 (8)
C8	0.0568 (13)	0.0435 (11)	0.0582 (14)	0.0003 (9)	0.0104 (11)	−0.0046 (9)
O1	0.0764 (12)	0.0545 (9)	0.0866 (13)	−0.0024 (8)	−0.0085 (10)	0.0031 (8)
C2	0.0626 (14)	0.0463 (12)	0.0608 (15)	0.0040 (10)	0.0063 (11)	−0.0061 (10)
C7	0.0694 (16)	0.0509 (12)	0.0679 (16)	−0.0083 (10)	0.0106 (13)	0.0044 (10)
C6	0.0761 (17)	0.0530 (13)	0.0683 (17)	−0.0025 (11)	0.0105 (13)	0.0114 (11)
C5	0.0677 (16)	0.0597 (13)	0.0713 (17)	0.0040 (12)	0.0086 (13)	0.0046 (11)
C4	0.0665 (16)	0.0555 (13)	0.0897 (19)	−0.0079 (11)	0.0045 (14)	0.0013 (13)
C3	0.0683 (15)	0.0418 (11)	0.0811 (17)	−0.0030 (10)	0.0059 (13)	0.0041 (10)
C1	0.0753 (19)	0.0700 (16)	0.091 (2)	−0.0008 (13)	−0.0140 (15)	−0.0020 (14)

### Geometric parameters (Å, °)

**Table:**

Cl1—C5	1.736 (3)	C7—H7A	0.9300
N1—C2	1.345 (3)	C6—C5	1.364 (4)
N1—C8	1.404 (3)	C6—H6A	0.9300
N1—H1A	0.8600	C5—C4	1.379 (3)
O2—C2	1.209 (3)	C4—C3	1.371 (3)
C8—C3	1.385 (3)	C4—H4A	0.9300
C8—C7	1.388 (3)	C3—H3A	0.9300
O1—C2	1.345 (3)	C1—H1B	0.9600
O1—C1	1.435 (3)	C1—H1C	0.9600
C7—C6	1.384 (4)	C1—H1D	0.9600
			
C2—N1—C8	128.2 (2)	C6—C5—C4	120.1 (2)
C2—N1—H1A	115.9	C6—C5—Cl1	120.07 (19)
C8—N1—H1A	115.9	C4—C5—Cl1	119.9 (2)
C3—C8—C7	118.6 (2)	C3—C4—C5	119.4 (2)
C3—C8—N1	117.20 (19)	C3—C4—H4A	120.3
C7—C8—N1	124.2 (2)	C5—C4—H4A	120.3
C2—O1—C1	116.3 (2)	C4—C3—C8	121.5 (2)
O2—C2—O1	123.8 (2)	C4—C3—H3A	119.3
O2—C2—N1	126.9 (2)	C8—C3—H3A	119.3
O1—C2—N1	109.2 (2)	O1—C1—H1B	109.5
C6—C7—C8	119.7 (2)	O1—C1—H1C	109.5
C6—C7—H7A	120.2	H1B—C1—H1C	109.5
C8—C7—H7A	120.2	O1—C1—H1D	109.5
C5—C6—C7	120.8 (2)	H1B—C1—H1D	109.5
C5—C6—H6A	119.6	H1C—C1—H1D	109.5
C7—C6—H6A	119.6		

### Hydrogen-bond geometry (Å, °)

**Table:**

*D*—H···*A*	*D*—H	H···*A*	*D*···*A*	*D*—H···*A*
N1—H1A···O2^i^	0.86	2.22	3.069 (2)	168

Symmetry codes: (i) −*x*+2, *y*−1/2, −*z*+3/2.
